# Supplementary material for: In silico analysis and comparison of the metabolic capabilities of different organisms by reducing metabolic complexity
Source: Microbiome. 2026 Feb 3;14:82. doi: 10.1186/s40168-025-02299-0 (PMC12964762; doi:10.1186/s40168-025-02299-0)
Supplement: Supplementary file 2 — Supplementary Material 1. [file 40168_2025_2299_MOESM1_ESM.docx]

Supplementary information for:

*in silico* analysis and comparison of the metabolic capabilities of different organisms by reducing metabolic complexity

**Author List**

Evangelia Vayena^1^, Meriç Ataman^1,2^, Vassily Hatzimanikatis^1^ *

^1^Laboratory of Computational Systems Biotechnology, École Polytechnique Fédérale de Lausanne, EPFL, Lausanne, Switzerland

^2^Present address: Computational and Systems Biology, Biozentrum, University of Basel, 4056 Basel, Switzerland

^*^Corresponding author, Email: vassily.hatzimanikatis@epfl.ch

# Supplementary Figures


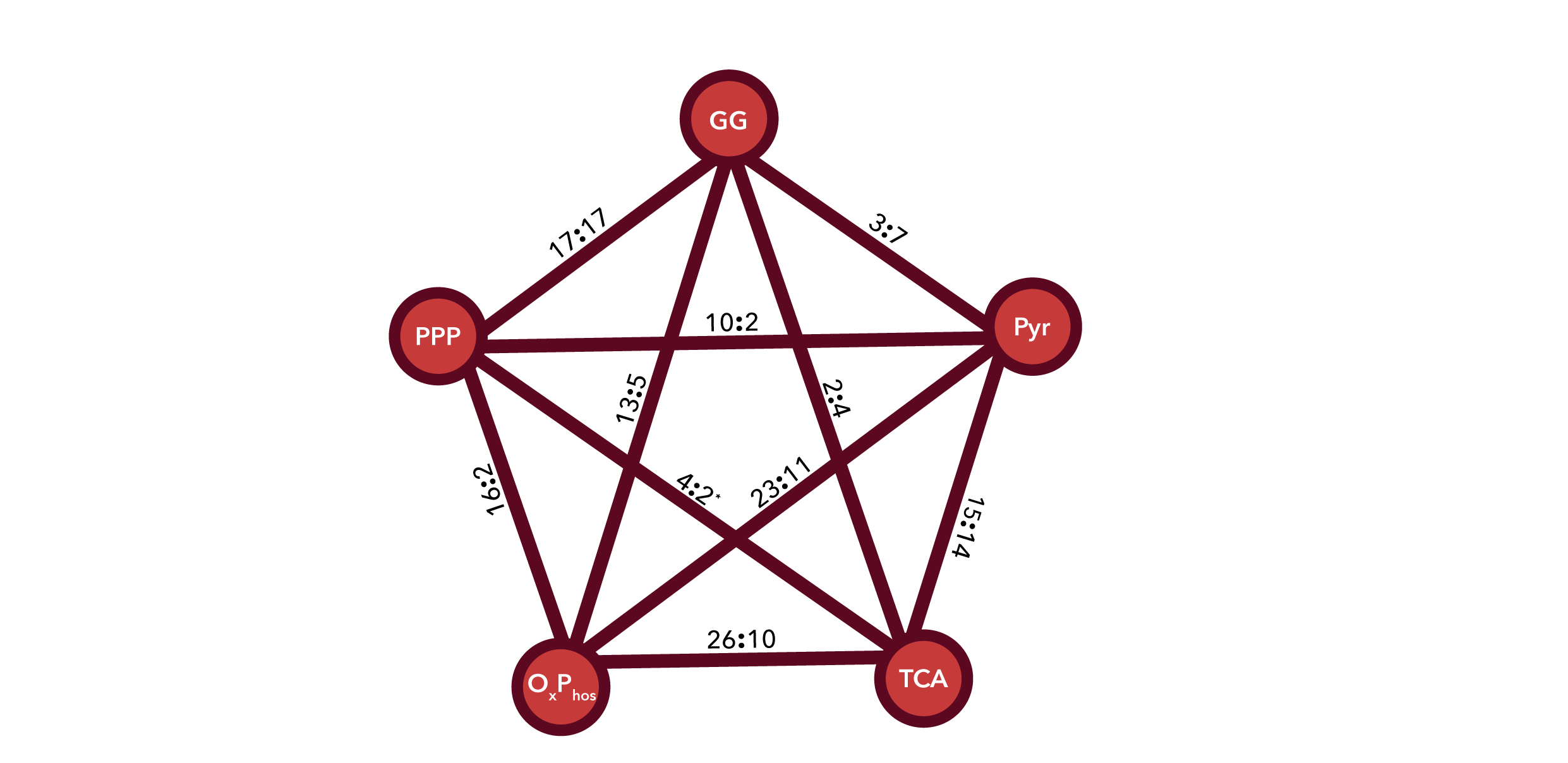


Fig S 1: Number of alternative pairwise connections between the initial metabolic subsystems in the D_1_ core network for E. coli : S. cerevisiae.

Fig S 2: The pentose phosphate pathway and TCA cycle subsystems and the D_1_ connections between them in the E. coli network. The plot is made with the Escher map web application^1^.

Fig S 3: The pentose phosphate pathway and TCA cycle subsystems and the D_1_ connections between them in the S. cerevisiae network. The plot is made with the Escher map web application^1^.


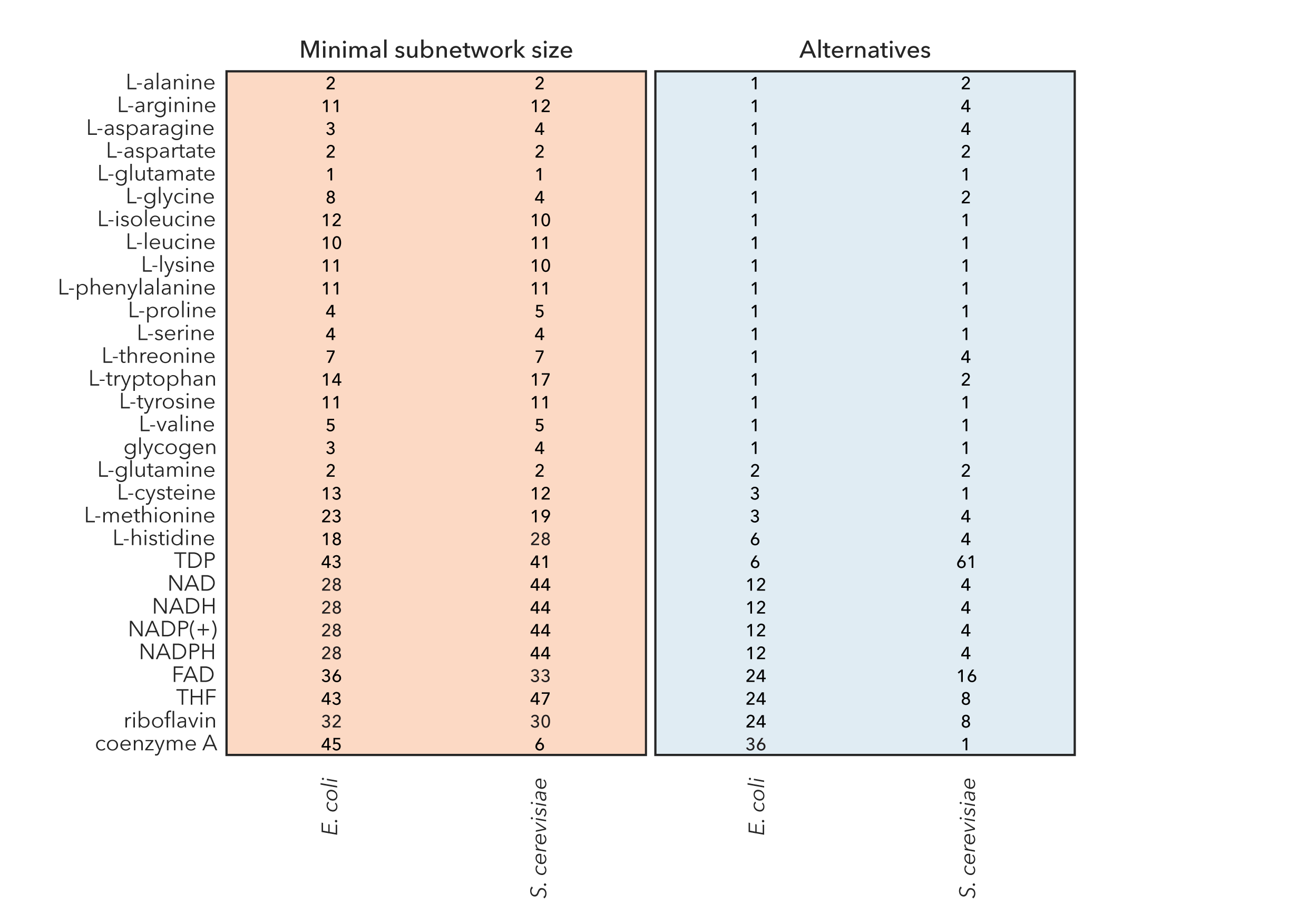


Fig S 4: The minimal subnetwork size for the biosynthesis of the common biomass building blocks in the E. coli and the S. cerevisiae networks and the number of alternative subnetworks of this size.


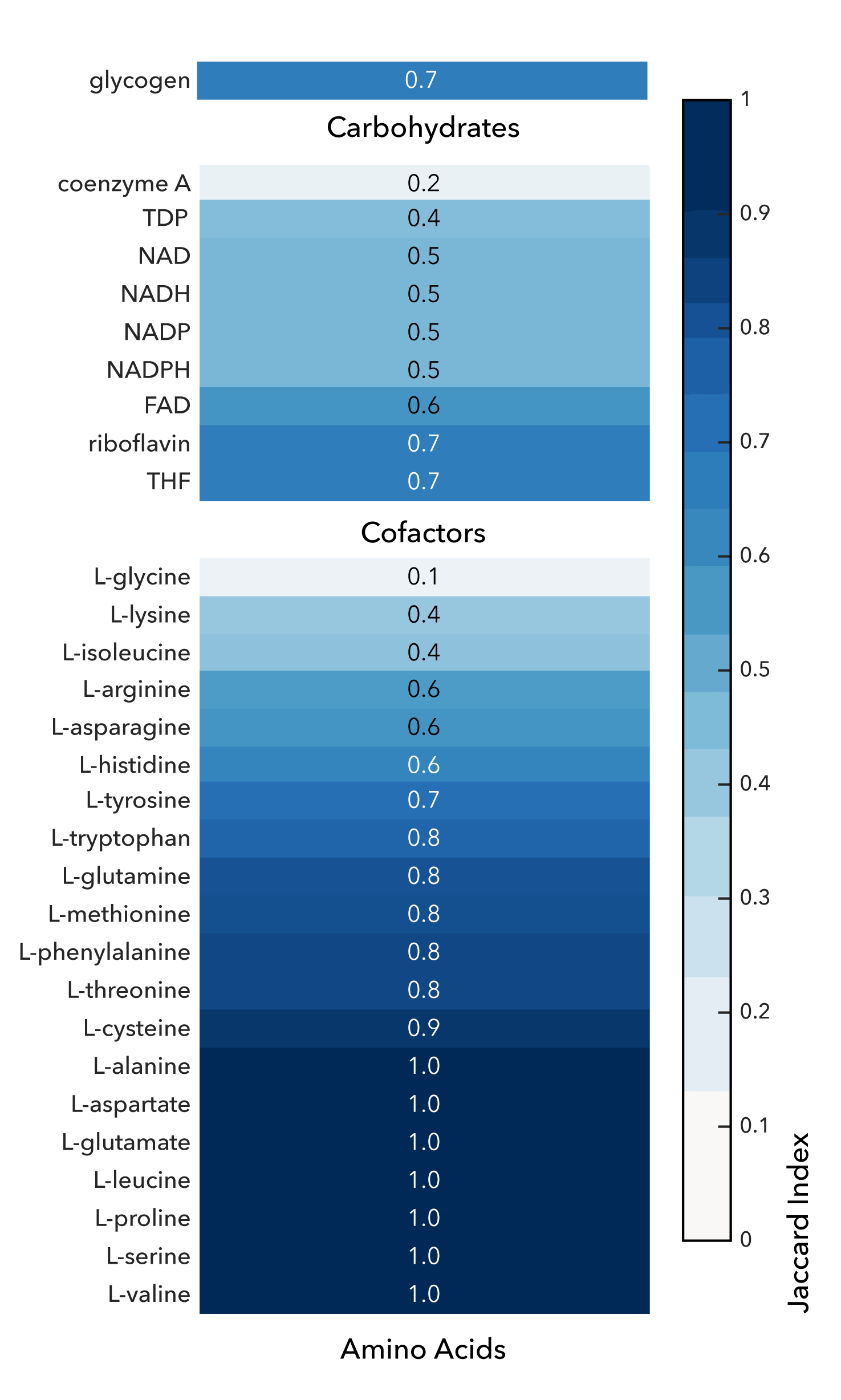


Fig S 5: Similarity of the cost of biosynthesis of the common biomass building blocks in the E. coli and the S. cerevisiae networks.

Fig S 6: Cost of biosynthesis of the amino acids for E. coli. The amino acids are sorted based on their relative abundance in the biomass.

Fig S 7: Cost of biosynthesis of the amino acids for S. cerevisiae. The amino acids are sorted based on their relative abundance in the biomass.


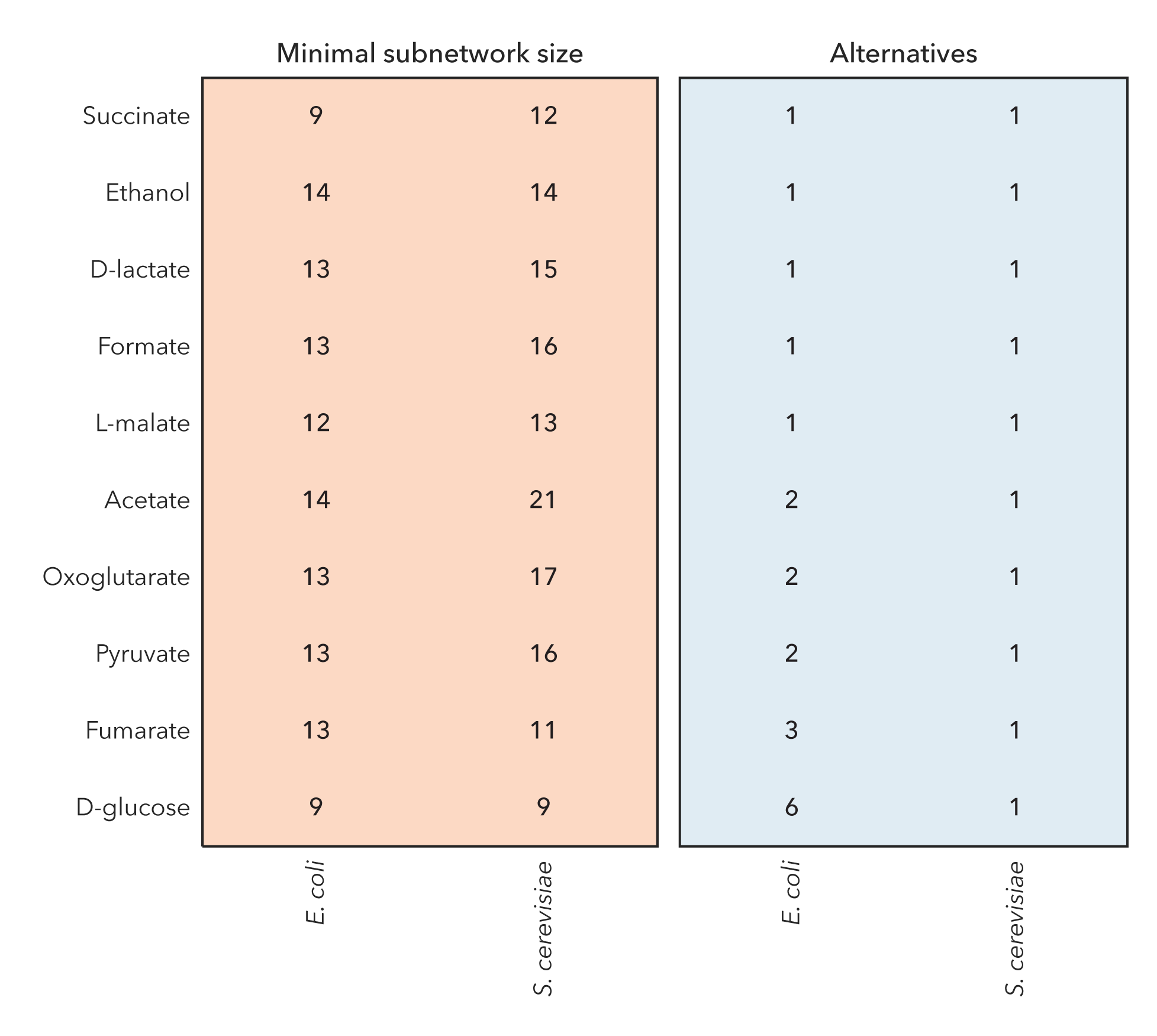


Fig S 8: The minimal subnetwork size for the uptake of ten selected compounds in the E. coli and the S. cerevisiae networks and the number of alternative subnetworks of this size.


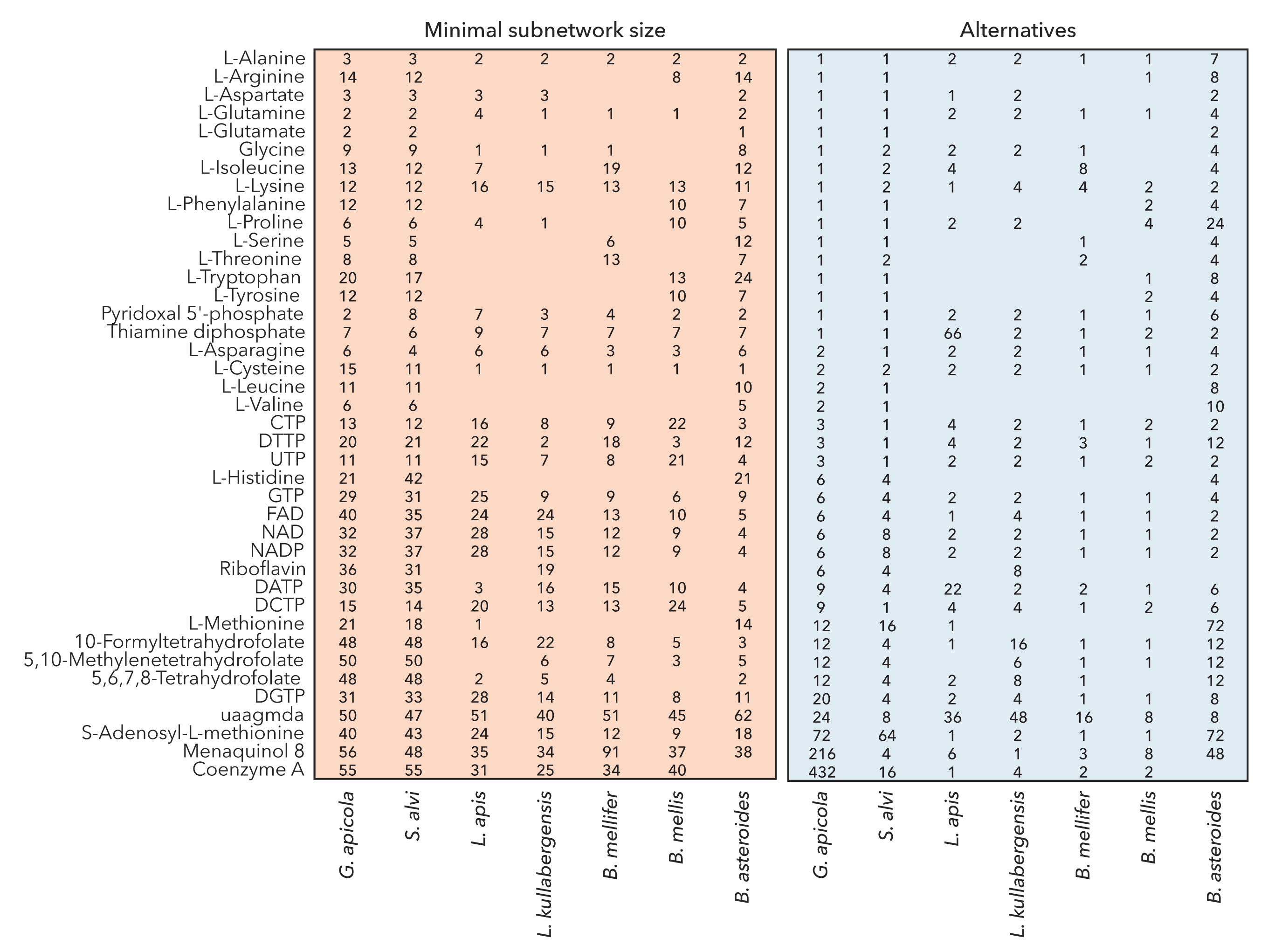

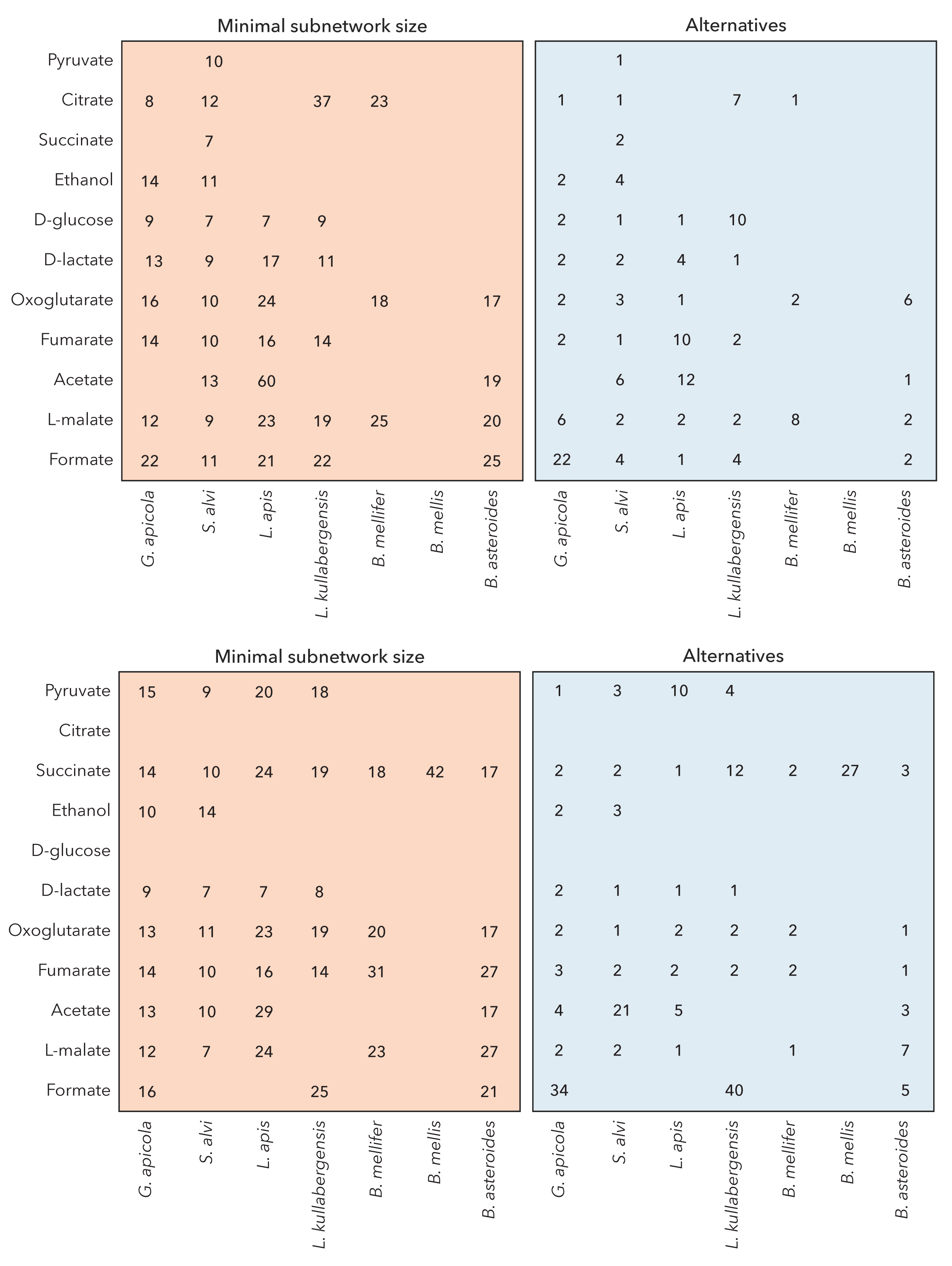


Fig S 9: The minimal subnetwork size for the biosynthesis of the biomass building blocks in the seven-member bee gut microbiome and the number of alternative subnetworks.

Fig S 10: The minimal subnetwork size for the uptake (up) and the secretion (down) of ten selected compounds in the seven-member bee gut microbiome and the number of alternative subnetworks.

# Supplementary Tables

Table S1: Number of metabolites (per model compartment, total and unique) in the D_0_ networks of E. coli and S. cerevisiae.

| Compartment in model | # of metabolites –  *E. coli* | # of metabolites –  *S. cerevisiae* |
| --- | --- | --- |
| Cytoplasm | 83 | 59 |
| Periplasm | 15 | - |
| Mitochondrion | - | 36 |
| Mitochondrial Membrane | - | 1 |
| Lipid Particle | - | 2 |
| Peroxisome | - | 14 |
| Endoplasmic Reticulum | - | 1 |
| Total | 98 | 113 |
| Unique | 87 | 71 |

*Table S2: The cost of biosynthesis of biosynthesis of the common biomass building blocks for E. coli and S. cerevisiae. The metabolite abbreviations follow the BiGG^2^ notation.*

BBB Cost of biosynthesis *E. coli* **:** Cost of biosynthesis *S. cerevisiae*

L-arginine 4 nh4 + 4 nadph + akg + accoa + 3 atp + oaa + co2 **:** 4 nh4 + 2.5 nadph + akg + 6.5 atp + hco3 + oaa + 1.5 nadh L-asparagine h + 2 nh4 + nadph + atp + oaa **:** 2 nh4 + 0.5 nadph + 2.5 atp + oaa + 0.5 nadh + 0.5 h2o

L-aspartate h + nh4 + nadph + oaa **:** nh4 + h + nadph + oaa

coenzyme A 7 nh4 + 11 nadph + 0.83 pyr + accoa + 19.67 atp + 3 oaa + 2 3pg + 2 nad + prpp + so4 + 2 for + 1.17 pep **:**

nh4 + nadph + akg

L-cysteine 2 h + nh4 + 5 nadph + accoa + 2.33 atp + 3pg + nad + so4 + 0.33 pep **:**

nh4 + h + 5 nadph + 2 atp + 3pg + accoa + nad + so4

FAD 10 nh4 + 4 nadph + 23.17 atp + 3 oaa + 2 co2 + 3 3pg + 4 nad + 2 prpp + 0.67 pep + 2 ru5p D + 6.5 h2o **:**

13 nh4 + 5.5 nadph + 40.06 atp + 4 oaa + 1.5 nadh + 8 h2o + 3 co2 + 4 ru5p D + 3 icit + 3 prpp + 0.06 fmn L-glutamine 2 nh4 + nadph + akg + 1.5 atp **:** 2 nh4 + 0.5 nadph + akg + 1.5 atp + 0.5 nadh

L-glutamate h + nh4 + nadph + akg **:** nh4 + h + nadph + akg

L-glycine nh4 + 3pg + nad + h2o **:** nh4 + h + nadph + icit glycogen atp + g6p **:** atp + h2o + g6p

L-histidine 3 nh4 + 2 nadph + 4.33 atp + oaa + 2 nad + prpp + for + 0.33 pep + h2o **:**

4 nh4 + 1.5 nadph + 5.5 atp + oaa + 1.5 h2o + 3pg + 1.5 nad + 2 prpp + o2

L-isoleucine 5 h + nh4 + 5 nadph + pyr + 2 atp + oaa :nh4 + 2 nadph + pyr + akg

L-leucine 2 h + nh4 + 2 nadph + 2 pyr + accoa + nad :nh4 + 2 h + 2 nadph + 2 pyr + accoa + nad

L-lysine 4 h + 2 nh4 + 4 nadph + pyr + atp + oaa + succoa **:** 2 nh4 + h + 4 nadph + akg + atp + accoa + 2 nad L-methionine 4 h + 2 nh4 + 9 nadph + accoa + 3.33 atp + oaa + 2 3pg + nad + so4 + 0.33 pep + succoa **:**

2 nh4 + 5 h + 8.5 nadph + 3 atp + oaa + 3pg + accoa + 0.5 nad + so4

NAD 7.5 nh4 + 3 nadph + 12.08 atp + 3 oaa + 1.5 3pg + 0.5 nad + 2 prpp + 0.5 for + 0.33 pep + dhap + iasp **:**

8 nh4 + 4 nadph + 17 atp + 2 oaa + h2o + 2 3pg + icit + 4 prpp + 4 o2 + e4p + 2 pep

NADH 7.5 nh4 + 3 nadph + 12.08 atp + 3 oaa + 1.5 3pg + 0.5 nad + 2 prpp + 0.5 for + 0.33 pep + dhap + iasp **:**

8 nh4 + 4 nadph + 17 atp + 2 oaa + h2o + 2 3pg + icit + 4 prpp + 4 o2 + e4p + 2 pep

*Continued on the next page*.

BBB Cost of biosynthesis *E. coli* **:** Cost of biosynthesis *S. cerevisiae*

NADP 7.5 nh4 + 3 nadph + 12.08 atp + 3 oaa + 1.5 3pg + 0.5 nad + 2 prpp + 0.5 for + 0.33 pep + dhap + iasp **:**

8 nh4 + 4 nadph + 17 atp + 2 oaa + h2o + 2 3pg + icit + 4 prpp + 4 o2 + e4p + 2 pep

NADPH 7.5 nh4 + 3 nadph + 12.08 atp + 3 oaa + 1.5 3pg + 0.5 nad + 2 prpp + 0.5 for + 0.33 pep + dhap + iasp **:**

8 nh4 + 4 nadph + 17 atp + 2 oaa + h2o + 2 3pg + icit + 4 prpp + 4 o2 + e4p + 2 pep L-phenylalanine nh4 + 2 nadph + atp + 2 pep + e4p :nh4 + 2 h + 2 nadph + atp + e4p + 2 pep

L-proline 2 h + nh4 + 3 nadph + akg + atp :nh4 + 3 h + 3 nadph + akg + atp

riboflavin 4.5 nh4 + 2 nadph + 11.5833 atp + oaa + co2 + 1.5 3pg + 2.5 nad + prpp + 0.33 pep + 2 ru5p D + 4.75 h2o **:**

13 nh4 + 5 nadph + 40 atp + 3 oaa + 11 h2o + 3 co2 + 4 ru5p D + 3 icit + 3 prpp

L-serine nh4 + nadph + 3pg + nad :nh4 + nadph + 3pg + nad

THF 7.5 nh4 + 4 nadph + akg + 16.08 atp + oaa + co2 + 1.5 3pg + 3.5 nad + prpp + 2.33 pep + 7.25 h2o + e4p **:**

11 nh4 + 5.5 nadph + akg + 31.5 atp + 2 oaa + 0.5 nadh + 9.5 h2o + 2 co2 + 2 ru5p D + 2 icit + 2 prpp + e4p + 2 pep TDP 5 nh4 + 9 nadph + 0.67 pyr + accoa + 13.83 atp + 2 3pg + 4 nad + 2 prpp + so4 + 2.33 pep + 2.5 h2o + e4p + g3p **:**

4 nh4 + 6.03 nadph + 13.47 atp + oaa + 2.52 3pg + 2 accoa + 0.83 nad + so4 + 2 icit + 2.52 prpp + 1.52 o2 + 0.51 r5p + 0.49 xu5p D

L-threonine 2 h + nh4 + 3 nadph + 2 atp + oaa :nh4 + 2 h + 2.5 nadph + 2 atp + oaa + 0.5 nadh

L-tryptophan 2 nh4 + nadph + 2 atp + prpp + 2 pep + e4p **:** 2 nh4 + 1.5 nadph + 2.5 atp + 3pg + 0.5 nad + prpp + e4p + 2 pep L-tyrosine nh4 + 2 nadph + atp + nad + 2 pep + e4p :nh4 + h + nadph + atp + e4p + 2 pep

L-valine 3 h + nh4 + 2 nadph + 2 pyr :nh4 + 3 h + 2 nadph + 2 pyr

*Table S3: The in silico defined minimal media for the core bee gut microbiome.*

| *G. apicola* | *S. alvi* | *L. apis* | *L. kullabergensis* | *B. mellifer* | *B. mellis* | *B. asteroides* |
| --- | --- | --- | --- | --- | --- | --- |
| D-Glucose | H2O | D-Glucose | D-Glucose | H2O | H2O | H2O |
| H2O | H+ | H2O | H2O | H+ | H+ | H+ |
| H+ | Chloride | H+ | H+ | L-Leucine | L-Leucine | Chloride |
| Chloride | Phosphate | L-Leucine | L-Leucine | Chloride | Chloride | Phosphate |
| Phosphate | Ammonium | Chloride | Chloride | Phosphate | Phosphate | Riboflavin |
| Ammonium | Fe3+ | Phosphate | Phosphate | Riboflavin | Riboflavin | Adenosine |
| Fe3+ | Potassium | Riboflavin | Ammonium | Ammonium | Ammonium | Ammonium |
| Potassium | Calcium | Aminoimidazole-riboside | L-Serine | L-Arginine | Guanine | L-Cysteine |
| Calcium | Citrate | L-Malate | L-Threonine | Cys-Gly | L-Serine | Potassium |
| Magnesium | Magnesium | L-Serine | L-Arginine | Fe3+ | L-Threonine | Calcium |
| Mn2+ | Mn2+ | L-Threonine | Cys-Gly | L-Aspartate | Cys-Gly | Magnesium |
| Co2+ | Co2+ | L-Arginine | Ornithine | L-Phenylalanine | Fe3+ | Mn2+ |
| Zinc | Zinc | Cys-Gly | L-Phenylalanine | Potassium | Ornithine | Co2+ |
| CMP | Cu2+ | Ornithine | Potassium | Beta-Alanine | L-Aspartate | Zinc |
| Cu2+ | O2 | L-Phenylalanine | Benzoate | Benzoate | Potassium | CMP |
| Nicotinate | Sulfate | Potassium | Calcium | Calcium | Beta-Alanine | Coenzyme A |
| Pyridoxal | Thiamin | Butyrate | Magnesium | Citrate | Calcium | Cu2+ |
| Sulfate |  | Benzoate | Mn2+ | Magnesium | Magnesium | Fe2+ |
| Thiamin |  | Calcium | Co2+ | Mn2+ | Mn2+ | D-Glucuronate |
|  |  | Magnesium | Zinc | Co2+ | Co2+ | Guanosine |
|  |  | Mn2+ | Cu2+ | Zinc | Zinc | Uaagmda |
|  |  | Co2+ | L-Glutamate | Cu2+ | Cu2+ | NMN |
|  |  | Zinc | DTMP | L-Glutamate | L-Glutamate | Shikimate |
|  |  | Cu2+ | Folate | Fe2+ | Fe2+ | Thiamin |
|  |  | L-Glutamate | Fumarate | Folate | Folate | Sulfate |
|  |  |  | *Continued on the next page*. |  |  |  |

| *G. apicola* | *S. alvi* | *L. apis* | *L. kullabergensis* | *B. mellifer* | *B. mellis* | *B. asteroides* |
| --- | --- | --- | --- | --- | --- | --- |
|  |  | Deoxyadenosine | L-Histidine | L-Histidine | Cellobiose | Folate |
|  |  | Folate | L-Isoleucine | L-Methionine | L-Histidine | D-Mannose 1-phosphate |
|  |  | L-Histidine | L-Methionine | NMN | L-Isoleucine |  |
|  |  | L-Methionine | NMN | Sulfate | L-Methionine |  |
|  |  | Nicotinate | (R)-Pantothenate | L-Tryptophan | NMN |  |
|  |  | (R)-Pantothenate | L-Tryptophan | L-Tyrosine | Sulfate |  |
|  |  | L-Tryptophan | L-Tyrosine | L-Valine | L-Valine |  |
|  |  | L-Tyrosine | D-Ribose | Thiamin | Shikimate |  |
|  |  | L-Valine | Thiamin | Uracil | Thiamin |  |
|  |  | Fe-enterobactin | Undecaprenyl-P | Xanthosine | Thymidine |  |
|  |  | Sulfate | Uracil |  |  |  |
|  |  | Thiamin | Xanthosine |  |  |  |
|  |  |  | Sulfate |  |  |  |
|  |  |  | Fe-enterobactin |  |  |  |
|  |  |  | Pyridoxal |  |  |  |
|  |  |  | L-Valine |  |  |  |

Table S4: The cost of biosynthesis of biosynthesis of the biomass building blocks for the core bee gut microbiome. The metabolite abbreviations follow the BiGG^2^ notation. Empty entry signifies that the BBB can be produced from the core network.

| BBB | Cost of biosynthesis *G. apicola* |
| --- | --- |
| 10fthf | 29 atp + 12 h2o + 8 nh4 + nad + 10 nadph + akg + co2 + 2 for + 2 pep + 3 oaa + r5p + e4p |
| ala L | atp + nh4 + nadph + pyr |
| amet | 22.5 atp + 4.5 h2o + 6 nh4 + 10.33 nadph + co2 + accoa + 3pg + 2 for + 3 oaa + r5p + so4 + 0.33 nad |
| arg L | 9 atp + 3 h2o + 4 nh4 + 4 nadph + akg + co2 + oaa |
| asn L | 3.5 atp + 1.5 h2o + 2 nh4 + nadph + oaa |
| asp L | atp + nh4 + nadph + oaa |
| coa | 14.75 atp + 6.75 h2o + nad + 5.5 nadph + 1.5 h_p + 1.5 h2o_p + pyr + 0.5 accoa + 3pg + for + oaa + 0.5 so4 + 1.5 cmp_p + nh4 |
| ctp | 6.667 atp + 3.67 h2o + h_p + h2o_p + cmp_p |
| cys L | 4 atp + nh4 + h + nad + 5 nadph + accoa + 3pg + so4 |
| datp | 19 atp + 3 h2o + 5 nh4 + 0.66 coa + 5.33 nadph + 0.66 pyr + 2 for + 3 oaa + r5p + 0.33 co2 |
| dctp | 6.667 atp + 2.667 h2o + 0.66 coa + h_p + h2o_p + 0.66 pyr + cmp_p + 0.33 nadph |
| dgtp | 19.5 atp + 5.5 h2o + 5 nh4 + nad + 4.4 nadph + 0.4 co2 + 2 for + 2 oaa + r5p + 0.6 coa + 0.6 pyr |
| dttp | 5.667 atp + 0.66 h2o + 1.33 h + 3 nadph + h_p + h2o_p + for + cmp_p |
| fad | 39.5 atp + 13.5 h2o + 9 nh4 + nad + 10 nadph + 2 co2 + for + 2 ru5p D + 5 oaa + 2 r5p |
| gln L | 2 atp + 2 nh4 + nadph + akg |
| glu L | atp + nh4 + nadph + akg |
| gly | 3 atp + h2o + nh4 + h + 3 nadph + oaa |
| gtp | 19.5 atp + 6.5 h2o + 5 nh4 + nad + 4 nadph + co2 + 2 for + 2 oaa + r5p |
| his L | 9 atp + 4 h2o + 3 nh4 + 2 nad + 2 nadph + for + oaa + r5p |
| ile L | 4 atp + nh4 + 3 h + 5 nadph + pyr + oaa |
| leu L | atp + nh4 + h + nad + 2 nadph + 2 pyr + accoa |
| lys L | 3 atp + 2 nh4 + 2 h + 4 nadph + pyr + succoa + oaa |
| met L | 6 atp + nh4 + 6 h + 0.66 nadh + 8.33 nadph + accoa + for + oaa + so4 |
| mlthf | 29 atp + 11 h2o + 8 nh4 + nad + 11 nadph + akg + co2 + 2 for + 2 pep + 3 oaa + r5p + e4p |
| mql8 | 146.67 atp + 84.67 h2o + 25.33 nadh + 24.67 nadph + 14 pyr + 2 succoa + 2 for + 4 pep + 2 oaa + 2 r5p + 2 e4p + 16 g3p |
| nad | 24.5 atp + 8.5 h2o + 6 nh4 + 5 nadph + co2 + 2 for + 3 oaa + 2 r5p + nac_p |
| nadp | 4.9 atp + 1.7 h2o + 1.2 nh4 + nadph + 0.2 co2 + 0.4 for + 0.6 oaa + 0.4 r5p + 0.2 nac_p |
| phe__L | 2 atp + nh4 + h + 2 nadph + 2 pep + e4p |
| pro L | 2 atp + nh4 + 2 h + 3 nadph + akg |
|  | *Continued on the next page*. |
| BBB | Cost of biosynthesis *G. apicola* |
| pydx5p | atp + pydx_p |
| ribflv | 19.5 atp + 8.5 h2o + 4 nh4 + nad + 5 nadph + co2 + 2 ru5p D + 2 oaa + r5p |
| ser L | atp + h2o + nh4 + nad + nadph + 3pg |
| thf | 28 atp + 12 h2o + 8 nh4 + nad + 10 nadph + akg + co2 + for + 2 pep + 3 oaa + r5p + e4p |
| thmpp | 4 atp + 2 h2o + thm |
| thr L | 3 atp + h2o + nh4 + h + 3 nadph + oaa |
| tyr__L | 2 atp + nh4 + nad + 2 nadph + 2 pep + e4p |
| trp L | 5 atp + 2 nh4 + nad + 2 nadph + 3pg + 2 pep + r5p + e4p |
| uaagmda | 100.33 atp + 52.33 h2o + 8 nh4 + 16.5 nadh + 20 nadph + 13.5 pyr + akg + succoa + 2 accoa + pep + 2.5 oaa + 2 f6p + 11 g3p |
| utp | 4.667 atp + 2.667 h2o + h_p + h2o_p + cmp_p |
| val__L | atp + nh4 + 2 h + 2 nadph + 2 pyr |
| BBB | Cost of biosynthesis *S. alvi* |
| 10fthf | 53.5 atp + 17.5 h2o + 15 nh4 + nad + 14 nadph + akg + 3 co2 + 3 prpp + 4 ru5p D + 2 pep + 6 oaa + e4p |
| ala L | atp + nh4 + nadph + pyr |
| amet | 53.5 atp + 4.5 h2o + 16 nh4 + 2 nad + 30 nadph + 3 co2 + 1.5 succoa + 3 3pg + 3 prpp + 4 ru5p D + 7 oaa + 3 so4 + 1.5 accoa |
| arg L | 8 atp + 2 h2o + 4 nh4 + 4 nadph + akg + co2 + oaa |
| asn L | 2 atp + 2 nh4 + nadph + oaa |
| asp L | atp + nh4 + nadph + oaa |
| coa | 46.75 atp + 11.25 h2o + 11.5 nh4 + nadh + 15.5 nadph + pyr + co2 + 0.5 accoa + 2.5 prpp + 4 ru5p D + 6.5 oaa + 0.5 so4 |
| ctp | 8 atp + 2 h2o + 3 nh4 + nadph + prpp + oaa + orot |
| cys L | 2 atp + nh4 + 2 h + nad + 5 nadph + accoa + 3pg + so4 |
| datp | 49 atp + 13 h2o + 13 nh4 + nadh + 13 nadph + 3 co2 + 3 prpp + 4 ru5p D + 7 oaa |
| dctp | 8 atp + h2o + 3 nh4 + 2 nadph + prpp + oaa + orot |
| dgtp | 49.5 atp + 14.5 h2o + 13 nh4 + 12 nadph + 3 co2 + 3 prpp + 4 ru5p D + 6 oaa |
| dttp | 7 atp + 3 nh4 + nad + 4 nadph + 3pg + prpp + oaa + orot |
| fad | 50 atp + 14 h2o + 13 nh4 + nadh + 12 nadph + 3 co2 + 3 prpp + 4 ru5p D + 7 oaa |
| gln L | 2 atp + 2 nh4 + nadph + akg |
| glu L | atp + nh4 + nadph + akg |
| gly | 3 atp + h2o + nh4 + h + nadh + 2 nadph + oaa |
| gtp | 49.5 atp + 15.5 h2o + 13 nh4 + 11 nadph + 3 co2 + 3 prpp + 4 ru5p D + 6 oaa |
| his L | 22.5 atp + 7.5 h2o + 7 nh4 + 2 nad + 6 nadph + co2 + 2 prpp + 2 ru5p D + 3 oaa |
|  | *Continued on the next page*. |
| BBB | Cost of biosynthesis *S. alvi* |
| ile L | 3 atp + nh4 + 3 h + nadh + 4 nadph + pyr + succoa + oaa |
| leu L | atp + nh4 + h + nad + 2 nadph + 2 pyr + accoa |
| lys L | 3 atp + 2 nh4 + 2 h + 4 nadph + pyr + succoa + oaa |
| met L | 4 atp + 2 nh4 + 5 h + 8 nadph + 0.5 succoa + 3pg + oaa + so4 + 0.5 accoa |
| mlthf | 53.5 atp + 16.5 h2o + 15 nh4 + nad + 15 nadph + akg + 3 co2 + 3 prpp + 4 ru5p D + 2 pep + 6 oaa + e4p |
| mql8 | 208 atp + 72 h2o + 4 nh4 + 44 nadh + 49 nadph + 28 pyr + 4 succoa + 4 3pg + 36 prpp + 8 pep + 4 e4p + 32 g3p |
| nad | 25.75 atp + 6.25 h2o + 7.5 nh4 + 0.5 nadh + 6.5 nadph + co2 + 2 prpp + 2 ru5p D + 4 oaa + 0.5 dhap + 0.5 iasp |
| nadp | 3.96 atp + 0.96 h2o + 1.15 nh4 + 0.08 nadh + nadph + 0.15 co2 + 0.31 prpp + 0.31 ru5p D + 0.61 oaa + 0.08 dhap + 0.08 iasp |
| phe L | 2 atp + nh4 + h + 2 nadph + 2 pep + e4p |
| pro L | 2 atp + nh4 + 2 h + 3 nadph + akg |
| pydx5p | atp + nh4 + 4 nad + nadph + pyr + e4p + g3p |
| ribflv | 16.5 atp + 5.5 h2o + 4 nh4 + 4 nadph + co2 + prpp + 2 ru5p D + 2 oaa |
| ser L | atp + h2o + nh4 + nad + nadph + 3pg |
| thf | 36 atp + 12 h2o + 11 nh4 + nad + 10 nadph + akg + 2 co2 + 2 prpp + 2 ru5p D + 2 pep + 4 oaa + e4p |
| thmpp | 4 atp + h2o + thm |
| thr L | 3 atp + h2o + nh4 + h + nadh + 2 nadph + oaa |
| trp L | 3 atp + 2 nh4 + nad + 2 nadph + 3pg + prpp + 2 pep + e4p |
| tyr L | 2 atp + nh4 + nad + 2 nadph + 2 pep + e4p |
| uaagmda | 83 atp + 23 h2o + 8 nh4 + 16.5 nadh + 20 nadph + 13.5 pyr + akg + succoa + 2 accoa + 11 prpp + pep + 2.5 oaa + 2 f6p + 11 g3p |
| utp | 6 atp + h2o + 2 nh4 + nadph + prpp + oaa + orot |
| val L | atp + nh4 + 2 h + 2 nadph + 2 pyr |
| BBB | Cost of biosynthesis *L. apis* |
| 10fthf | 3.5 atp + 3.5 h2o + 0.5 h + 0.5 nadh + nadph + 0.5 pyr + 0.5 airs_p + 2.5 cgly + 0.5 g3p + fol + 0.5 tyr L |
| ala L | h2o + cgly |
| amet | 8.5 atp + 2 glu L + 8 h2o + 0.5 nad + gtp + 3 ser L + 2 oaa + 1.5 airs_p + 5 cgly + 0.5 g3p + 0.5 tyr L + met L_p |
| arg L |  |
| asn L | 2 atp + glu L + h2o + ser L + oaa |
| asp L | glu L + ser L + oaa |
| coa | 10.5 atp + 2 glu L + 11 h2o + 0.5 nad + gtp + 3 ser L + 2 oaa + 1.5 airs_p + 9 cgly + utp + 0.5 g3p + 0.5 tyr L + pnto R |
| ctp | 7.5 atp + 2 glu L + 2.5 h2o + 2 ser L + oaa + r5p + utp + orot |
| cys L | h2o + cgly |
|  | *Continued on the next page*. |
| BBB | Cost of biosynthesis *L. apis* |
| datp | 3 atp + dad_2 |
| dctp | 8.5 atp + 2 glu L + 2.5 h2o + nadph + 2 ser L + oaa + r5p + utp + orot |
| dgtp | 9.5 atp + 8.5 h2o + 0.5 nad + nadph + 2 ser L + oaa + 1.5 airs_p + 8.5 cgly + 0.5 g3p + 0.5 tyr L |
| dttp | 8.5 atp + 3 glu L + 2 nadph + 3 ser L + oaa + r5p + orot + h2o + 0.5 utp |
| fad | 8.5 atp + 2 glu L + 8 h2o + 0.5 nad + gtp + ribflv + 3 ser L + 2 oaa + 1.5 airs_p + 7 cgly + 0.5 g3p + 0.5 tyr L |
| gln L | atp + 2 glu L + ser L + oaa |
| glu L |  |
| gly | h2o + cgly |
| gtp | 17 atp + 18.5 h2o + nad + 4 ser L + 2 oaa + 3 airs_p + 16 cgly + g3p + tyr L + 0.5 pep |
| his L |  |
| ile L | 3 h + nadh + nadph + pyr + thr L |
| leu L |  |
| lys L | atp + 3 glu L + 2 h2o + 3 ser L + oaa + actp |
| met L | atp + h2o + met L_p |
| mlthf |  |
| mql8 | 460 atp + 6 glu L + 432 h2o + 455 h + 72 nad + 102 nadph + 16 gtp + 6 succoa + 11 fad + 96 ser L + 240 crn + 72 but + 6 bz + 120 feenter_p + 6 met L_p + 16 utp |
| nad | 23 atp + 24 h2o + 2 gtp + 2 ser L + 4 oaa + 2 r5p + 3 airs_p + 18 cgly + g3p + tyr L + 2 nac |
| nadp | 46 atp + 52 h2o + 5 gtp + 4 ser L + 8 oaa + 4 r5p + 6 airs_p + 40 cgly + 2 g3p + 2 tyr L + 4 nac |
| phe L |  |
| pro L | h + nadh + pyr + orn |
| pydx5p | atp + 3 h2o + nad + r5p + 5 cgly + g3p |
| ribflv |  |
| ser L |  |
| thf | 2 h + 2 nadph + fol |
| thmpp | 4.33 atp + 3 h2o + 0.33 gtp + thm_e + 0.33 utp |
| thr L |  |
| trp L |  |
| tyr L |  |
| uaagmda | 154 atp + 56.33 glu L + 139 h2o + 85.67 h + 18 nad + 3.67 gtp + 45 ser L + pep + oaa + 72 crn + 2 f6p + 18 but + 5.67 utp + 36 feenter_p |
| utp | 14 atp + 6 glu L + 4 h2o + 4 ser L + 2 oaa + 2 r5p + 2 orot + pep |
| val L |  |
|  | *Continued on the next page*. |
| BBB | Cost of biosynthesis *L. kullabergensis* |
| 10fthf | 4 atp + 1.67 h2o + 0.67 h + 2.33 nadph + 0.67 ru5p D + 1.17 oaa + 0.67 rib D + fol + 2.33 trp L + 0.33 xtsn + 1.17 pyr |
| ala__L | h2o + cgly |
| amet | 8 atp + 5 h2o + nadph + oaa + met L + trp L + xtsn |
| arg L |  |
| asn L | 2 atp + 2 nh4 + nadh + oaa |
| asp L | nh4 + h + nadh + oaa |
| coa | 12 atp + 13457.6 h2o + nadph + oaa + 13452.6 cgly + trp L + xtsn + pnto R |
| ctp | 6 atp + h2o + nh4 + rib D + ura |
| cys L | h2o + cgly |
| datp | 8 atp + 2 h2o + 2 nadph + 2 oaa + 2 trp L + xtsn |
| dctp | 6 atp + nh4 + nadph + 0.5 pyr + rib D + ura + trp L + 0.5 oaa |
| dgtp | 7 atp + 2 h2o + nh4 + nadph + 0.5 pyr + trp L + xtsn + 0.5 oaa |
| dttp | 2 atp + h + dtmp |
| fad | 18 atp + 9 h2o + 2 nadph + 2 ru5p D + 2 oaa + 2 rib D + 2 trp L + 2 xtsn |
| gln L | atp + glu L + nh4 |
| glu L |  |
| gly | h2o + cgly |
| gtp | 7 atp + 3 h2o + nh4 + xtsn |
| his L |  |
| ile L |  |
| leu L |  |
| lys L | atp + 4 h + 2 nadh + pyr + oaa + actp + 2 trp L |
| met L |  |
| mlthf | 4 h + 2 nadph + 0.67 pyr + ser L + fol + 2 trp L + 1.33 oaa |
| mql8 | 31 atp + 62.5 h2o + 35.5 nad + 16 nadph + 11.5 pyr + succoa + ser L + 30 glyc3p + bz + 12.5 val L + fum |
| nad | 8 atp + 4 h2o + nadph + oaa + nmn + trp L + xtsn |
| nadp | 8 atp + 4 h2o + nadph + oaa + nmn + trp L + xtsn |
| phe L |  |
| pro L | orn |
| pydx5p | atp + pydx_e |
| ribflv | 9 atp + 5 h2o + nadph + 0.5 pyr + 2 ru5p D + 2 rib D + trp L + xtsn + 0.5 oaa |
|  | *Continued on the next page*. |
| BBB | Cost of biosynthesis *L. kullabergensis* |
| ser L |  |
| thf | 4 h + 2 nadph + pyr + fol + 2 trp L + oaa |
| thmpp | 4 atp + 2 h2o + thm |
| thr L |  |
| trp L |  |
| tyr L |  |
| uaagmda | 11 atp + glu L + 92.36 nh4 + 90.36 nadh + 90.36 pyr + pep + udcpp + oaa + actp + 2 f6p + val L + 81.36 h |
| utp | 5 atp + h2o + rib D + ura |
| val__L |  |
| BBB | Cost of biosynthesis *B. mellifer* |
| 10fthf | 2 h2o + h + nad + nadph + cgly + fol |
| ala L | h2o + cgly |
| amet | 8.1 atp + 5.1 h2o + nadph + asp L + met L + 1.1 xtsn |
| arg L |  |
| asn L | 2 atp + h2o + nh4 + asp L |
| asp L |  |
| coa | 19.30 atp + 8.30 h2o + 3 nadph + pyr + asp L + actp + ala_B + cgly + ura + val L + 2.3 xtsn + 0.5 nadh |
| ctp | 6 atp + 2 h2o + nh4 + ura + xtsn |
| cys L | h2o + cgly |
| datp | 8.2 atp + 2.2 h2o + 2 nadph + asp L + 1.2 xtsn |
| dctp | 7.1 atp + 2.1 h2o + nh4 + nadph + 1.1 xtsn |
| dgtp | 7.1 atp + 2.1 h2o + nh4 + nadph + 1.1 xtsn |
| dttp | 5.1 atp + 2.1 h2o + 1.67 nadph + cgly + ura + 1.1 xtsn + 0.67 nad |
| fad | 9.1 atp + 4.1 h2o + nadph + asp L + ribflv + 1.1 xtsn |
| gln L | atp + glu__L + nh4 |
| glu L |  |
| gly | h2o + cgly |
| gtp | 7 atp + 3 h2o + nh4 + xtsn |
| his L |  |
| ile L | 2.6 atp + glu L + 5.8 h + 4.5 nadh + 2.5 nadph + 2 pyr + asp L + 2 actp + 0.6 xtsn |
| leu L |  |
|  | *Continued on the next page*. |
| BBB | Cost of biosynthesis *B. mellifer* |
| lys L | 1.3 atp + glu L + 0.3 h2o + 2.9 h + 1.5 nadh + 2 nadph + pyr + asp L + 2 actp + 0.3 xtsn |
| met L |  |
| mlthf | 4 h2o + 2 h + 2 nad + 2 nadph + 3 cgly + fol |
| mql8 | 51.14 atp + glu L + 29.59 h2o + 2.21 nad + 19.88 nadph + 0.07 h2o_p + pyr + 24 actp + 1.14 cgly + bz + 2.74 ura + 6.80 xtsn + 1.59 nh4 |
| nad | 8.1 atp + 4.1 h2o + nadph + asp L + nmn + 1.1 xtsn |
| nadp | 8.1 atp + 4.1 h2o + nadph + asp L + nmn + 1.1 xtsn |
| phe L |  |
| pro L |  |
| pydx5p | 2.6 atp + nh4 + g3p + 1.60 xtsn |
| ribflv |  |
| ser L | 3 h2o + nad + nadph + 2 cgly |
| thf | 0.2 atp + 0.2 h2o + 1.6 h + 2 nadph + fol + 0.2 xtsn |
| thmpp | 4 atp + 2 h2o + thm |
| thr L | 2.2 atp + 1.2 h2o + 0.6 h + 1.5 nadph + asp L + 0.2 xtsn + 0.5 nadh |
| trp L |  |
| tyr L |  |
| uaagmda | 53 atp + 4 glu L + 35 h2o + nh4 + 0.5 nad + 25.5 nadph + 4 pyr + asp L + pep + 36 actp + 2 f6p + 2 cgly + 8 xtsn |
| utp | 5 atp + 2 h2o + ura + xtsn |
| val__L |  |
| BBB | Cost of biosynthesis *B. mellis* |
| 10fthf | h2o + nad + nadp + gly + thf |
| ala L | h2o + cgly |
| amet | 5 atp + 3 h2o + nadph + asp L + r5p + met L + gua |
| arg L | 5 atp + 3 h2o + nh4 + co2 + asp L + orn |
| asn L | 2 atp + h2o + nh4 + asp L |
| asp L |  |
| coa | 13 atp + 6.5 h2o + 1.5 nad + 3 nadph + 2 h_p + asp L + pep + 2 r5p + cgly + ala_B_p + utp + val L + gua + skm_p |
| ctp | 11 atp + 4 glu L + 0.5 nad + 4 nadph + r5p + utp + thymd |
| cys L | h2o + cgly |
| datp | 5 atp + 2 nadph + asp L + r5p + gua |
| dctp | 11 atp + 4 glu L + 0.5 nad + 5 nadph + r5p + utp + thymd |
|  | *Continued on the next page*. |
| BBB | Cost of biosynthesis *B. mellis* |
| dgtp | 4 atp + nadph + r5p + gua |
| dttp | 3 atp + thymd |
| fad | 6 atp + 2 h2o + nadph + asp L + ribflv + r5p + gua |
| gln L | atp + glu__L + nh4 |
| glu L |  |
| gly |  |
| gtp | 4 atp + h2o + r5p + gua |
| his _L |  |
| ile _L |  |
| leu L |  |
| lys__L | atp + 3 h + 2.5 nadph + pyr + asp L + actp + orn + 0.5 nadh |
| met__L |  |
| mlthf | nad + gly + thf |
| mql8 | 26 atp + glu L + 15.5 h2o + 24.5 nad + 16 nadph + 2 h_p + 11 acald + 2 pep + r5p + 2dmmql8 + 4 utp + thymd + 2 skm_p + 11 pyr |
| nad | 5 atp + 2 h2o + nadph + asp L + r5p + nmn + gua |
| nadp | 5 atp + 2 h2o + nadph + asp L + r5p + nmn + gua |
| phe L | atp + 0.5 nadph + h_p + pep + orn + skm_p + 0.5 nadh |
| pro L | atp + h_p + pep + orn + skm_p + 0.5 nadph |
| pydx5p | atp + nh4 + r5p + g3p |
| ribflv |  |
| ser L |  |
| thf |  |
| thmpp | 3.5 atp + 2 h2o + 0.5 utp + thm |
| thr L |  |
| trp L | 4 atp + nh4 + h_p + ser__L + pep + r5p + skm_p |
| tyr__L | atp + 0.5 nad + 0.5 nadph + h_p + pep + orn + skm_p |
| uaagmda | 36.5 atp + glu L + 26 h2o + 2 nh4 + 34.5 nad + 25.5 nadph + 18.5 pyr + asp L + 17.5 acald + pep + actp + orn + 2 f6p + 3 cgly + 7.5 utp |
| utp | 40 atp + 16 glu L + 3 h + 2 nad + 16 nadph + 4 r5p + + 4 thymd + pep |
| val L |  |
| BBB | BBB Cost of biosynthesis *B. asteroides* |
| 10fthf | atp + 2 h + 2 nadph + for + fol |
|  | *Continued on the next page*. |
| BBB | BBB Cost of biosynthesis *B. asteroides* |
| ala__L | nh4 + h + nadph + pyr |
| amet | 6 atp + 0.33 h2o + 2.67 h + 1.17 nadh + 5.5 nadph + adn + succoa + for + 2.67 oaa + cys__L_p + 1.67 nh4 |
| arg__L | 7.5 atp + 4 nh4 + 4 nadph + akg + co2 + oaa + 1.5 h2o |
| asn__L | 3.5 atp + 1.5 h2o + 2 nh4 + nadph + oaa |
| asp__L | nh4 + h + nadph + oaa |
| coa |  |
| ctp | 2 atp + h + cmp |
| cys__L | atp + h2o + cys__L_p |
| datp | 3 atp + h + nadph + adn |
| dctp | 2 atp + 2 h + nadph + cmp |
| dgtp | 7 atp + 4 h2o + nad + nadph + adn |
| dttp | 4 atp + 5.5 h + 3 nadph + for + 1.5 cmp |
| fad | 4 atp + h2o + adn + ribflv |
| gln__L | 1.5 atp + 2 nh4 + nadph + akg |
| glu__L | nh4 + h + nadph + akg |
| gly | 2 atp + 3 nh4 + 4 h + 0.5 nadh + 4.5 nadph + 3 oaa |
| gtp | 7 atp + 5 h2o + nad + adn |
| his__L | 8 atp + 3 h2o + 3 nh4 + 2 nad + 2 nadph + for + oaa + r5p |
| ile__L | 2 atp + 8.5 nh4 + 12.5 h + 12 nadph + pyr + 8.5 oaa + 0.5 nadh |
| leu__L | nh4 + 2 h + nad + 2 nadph + 2 pyr + accoa |
| lys__L | atp + 8 nh4 + 10 h + 10 nadph + pyr + succoa + 7 oaa |
| met__L | 3 atp + 4.67 h + 1.16667 nadh + 5.5 nadph + succoa + for + 2.67 oaa + cys__L_p + 1.67 nh4 |
| mlthf | atp + 3 h + 3 nadph + for + fol |
| mql8 | 1536 atp + 192 h2o + 480 h + 608 nadh + 448 nadph + 335 pyr + 48 akg + 48 for + 48 pep + 48 r5p + 384 g3p + 48 skm + lac__L |
| nad | 3 atp + h2o + adn + nmn |
| nadp | 7 atp + 2 h2o + 2 adn + 2 nmn |
| phe__L | atp + nh4 + h + nadph + pep + skm |
| pro__L | atp + nh4 + 3 h + 2 nadph + akg + nadh |
| pydx5p | atp + nh4 + r5p + g3p |
| ribflv |  |
| ser__L | 3 atp + 4.5 nh4 + 6.5 h + 0.5 nadh + 7 nadph + for + 4.5 oaa |
| thf | 2 h + 2 nadph + fol |
| thmpp | 4 atp + 2 h2o + thm |
| thr__L | 2 atp + 3 nh4 + 4 h + 4.5 nadph + 3 oaa + 0.5 nadh |
| trp__L | 11 atp + 9 nh4 + 0.5 nadh + 10.5 nadph + for + pep + 8 oaa + r5p + skm + 2 h |
| tyr__L | atp + nh4 + nad + nadph + pep + skm |
| uaagmda |  |
| utp | 2 atp + h2o + 2 h + cmp |
| val__L | nh4 + 3 h + 2 nadph + 2 pyr |

# References

1. Zachary A. King, Andreas Dräger, Ali Ebrahim, Nikolaus Sonnenschein, Nathan E. Lewis, and Bernhard O. Palsson. Escher: A Web Application for Building, Sharing, and Embedding Data- Rich Visualizations of Biological Pathways. *PLOS Computational Biology*, 11(8):e1004321, 8 2015. ISSN 1553-7358. doi: 10.1371/JOURNAL.PCBI.1004321. URL [https://journals.plos.org/](https://journals.plos.org/ploscompbiol/article?id=10.1371/journal.pcbi.1004321) [ploscompbiol/article?id=10.1371/journal.pcbi.1004321](https://journals.plos.org/ploscompbiol/article?id=10.1371/journal.pcbi.1004321).
2. Zachary A. King, Justin Lu, Andreas Dräger, Philip Miller, Stephen Federowicz, Joshua A. Lerman, Ali Ebrahim, Bernhard O. Palsson, and Nathan E. Lewis. BiGG Models: A platform for integrating, standardizing and sharing genome-scale models. *Nucleic Acids Research*, 44(D1):D515–D522, 1 2016. ISSN 13624962. doi: 10.1093/nar/gkv1049. URL <http://bigg.ucsd.edu/models/e>.
